# Determinants of defaulter to full vaccination among children aged 12–23 months in Siraro district, West Arsi zone, Oromia, Ethiopia: a case-control study

**DOI:** 10.1186/s12887-023-04029-7

**Published:** 2023-05-09

**Authors:** Ararso Hordofa Guye, Tadesse Nigussie, Mengistu Tesema, Dame Banti Shambi, Berhanu Senbeta Deriba, Negasa Shuma Dureso, Sisay Motuma Debela

**Affiliations:** 1 Department of Public Health, College of Medicine and Health Sciences, Salale University, Fiche, Ethiopia; 2 Department of Pediatrics and Child Health, College of Medicine and Health Sciences, Salale University, Fiche, Ethiopia

**Keywords:** Determinants, Defaulter, Full vaccination, Siraro District, West Arsi Zone

## Abstract

**Background:**

Vaccination is one of the most cost-effective global public health interventions to reduce childhood morbidity and mortality. Defaulters to full vaccination can put children at greater risk of acquiring vaccine-preventable disease outbreaks. The reason for not receiving full vaccination is not well explored, and hence, there is limited evidence about defaulters of vaccination in Ethiopia.

**Objectives:**

To identify determinants of defaulter to full vaccination among children aged 12–23 months in Siraro District, West Arsi Zone, Oromia Region, Ethiopia.

**Methods:**

A community-based unmatched case-control study was conducted among children aged 12–23 months from March 20 to April 30, 2022, with a total sample size of 444 (148 cases and 296 controls). Cases were children aged 12–23 months who had missed at least one routine vaccination dose, while controls were children who had received all of the recommended routine vaccinations. Consecutive sampling and simple random sampling techniques were used to select representative cases and controls respectively. Data were collected using a structured questionnaire, entered into Epi-data version 4.6, and exported to Statistical Package for Social Sciences version 26 for analysis. Logistic regression was used to identify determinants of the defaulter to full vaccination and the variables with p-value < 0.25 were recruited for multivariable analysis, and an adjusted odds ratio with a 95% confidence interval and a p-value of ≤ 0.05 was used to declare the statistical significance of the association.

**Result:**

Of the assessed determinants of the defaulter to full vaccination; inadequate knowledge of mothers/caretakers (AOR = 4.32, 95% CI:2.78–6.70), educational status of a father unable to read and write (AOR = 3.66, 95% CI:1.29–10.39), time to reach health facility ≥ 30 minutes (AOR = 2.45, 95% CI:1.51–3.97), not told about the type of vaccine received (AOR = 2.37, 95% CI;1.27–4.45), no parents discussion on vaccination (AOR = 2.16, 95% CI:1.24–3.79), home delivery (AOR = 2.43, 95% CI:1.39–4.25) and not participated in pregnant mother conference (AOR = 2.47, 95% CI = 1.35–4.49) were the identified determinants of the defaulter to full vaccination.

**Conclusion:**

Mother’s’ knowledge, father’s education, place of delivery, time to reach a health facility, health workers who told the type of vaccine received, participation in pregnant mother conference, and parents’ discussion on vaccination were the determinants of the defaulter to full vaccination status. Thus, the district health office should work on defaulters of vaccination by strengthening immunization service delivery and improving maternal knowledge on vaccination through pregnant mother conference participation.

## Introduction

Vaccination is the process of inducing a protective response in an individual’s body against a specific illness by administering vaccines and one of the most cost-effective global public health interventions to reduce childhood morbidity and mortality [1, 2]. Defaulter to full vaccination is a condition in which a child missed a routine vaccination schedule and does not become fully vaccinated for any reason that can put children at greater risk of acquiring vaccine-preventable diseases [3, 4]. Expanding universal age-specific immunization program is one of the areas where preventive public health policy has become successful [3]. Currently, the EPI has emerged as a highly effective and efficient method to improve child survival and the program’s interventions that led to a notable reduction in vaccine-preventable diseases worldwide [3, 5].

Globally, vaccine-preventable diseases are still the most common causes of childhood mortality with an estimated 3 million deaths every year, mainly in Africa and Asia [6]. In 2019, about 21.8 million eligible children did not receive 3 doses of the diphtheria, pertussis, and tetanus vaccine (DTP3); among them, 9.6 million (44%) started, but did not complete the DPT 3-dose series [7]. Partial child vaccination against vaccine-preventable diseases is a significant public health challenge. During this EPI era, children still die of vaccine-preventable diseases every year in many developing countries [5, 8]. Immunizations have brought comprehensive health to many children and have been concrete in protecting children against vaccine-preventable diseases (VPDs) in low-and middle-income countries [9, 10].

Even though Africa has achieved remarkable progress in immunization services, large numbers of children remain unvaccinated and under-vaccinated [5, 6]. Sub-Saharan African countries still there is the highest child mortality rate in the world and the region had an average child mortality rate of 76 deaths per 1000 live births in 2019 [9]. In most of these countries, poor functioning health service delivery systems, difficult topography, and armed conflict prevent the efforts to meet immunization targets, especially for children living in hard-to-reach areas [4]. Most children die because they do not access effective interventions that would combat common and preventable childhood illnesses [11, 12].

According to the Ethiopian Federal Ministry of Health vaccination schedule, full vaccination is considered as if the child has received one dose of Bacillus Calumet Guerin (BCG) vaccination against tuberculosis at birth or at the first clinical contact, three doses of pentavalent vaccine (DPT-HepB -Hib), three doses of the polio vaccine, three dose of pneumococcal conjugate vaccine (PCV) at age of 6, 10, and 14 weeks. In addition, two dose of rotavirus vaccine at age of 6 and 10 weeks and one dose of measles vaccine at 9 months or soon before their first birth year and the vaccinations be recorded on a vaccination card that is given to the parents or caretakers [10, 13, 14].

In Ethiopia, full vaccination against vaccine-preventable diseases are still a significant and persistent public health challenge [4, 15]. Expanding immunization service is among one of the Ethiopian child survival strategies targeted to protect an estimated 2 million annual childbirths from VPDs but a significant portion of children were not been vaccinated. As a result, children in the first year of life in Ethiopia are among the highest rate of acquiring VPDs. Many of these reasons were believed to be due to defaulters to full vaccination [8, 15].

The full vaccination coverage has been raised from 39% to 2016 to 43% in the 2019 EDHS report and about 19% of children in the age group of 12–23 months have not received any vaccinations at all [16]. The highest coverage of full vaccination in Addis Ababa (83%) and lowest in Afar region due to different challenges and opportunities as explained in other studies (20%) [7, 13]. The acceptable defaulter rate is less than 5% in Ethiopia and the defaulter rate with the cut-off point of more than 10% indicates immunization service utilization problem [8]. Studies have also reported that maternal education, place of delivery, antenatal care (ANC) visits, postnatal care follow-up, place of residence, and exposure to mass media as predictors of poor child immunization status [13, 14, 17, 18, 19].

However, most of the studies were cross-sectional and failed to include certain variables or factors that could be pertinent in the Ethiopian context, such as socioeconomic status, attitude, and service satisfaction towards immunization services provision at the grassroots of individual family members and only few community-based studies have been done. In the study area, defaulter to childhood vaccination is a critical problem and determinants of the defaulter to full vaccinations were not well known. Still, in the presence of vaccination service, some of the children were not become fully vaccinated throughout the year. According to the Siraro District DHIS-2 report in 2021/2022, the full vaccination status of the children was 87.8% and the reason for the rest was unknown. In addition, there are frequent vaccine-preventable disease outbreaks such as Polio and Measles. Due to these unknown factors and limited evidence about defaulters of childhood vaccination, identifying determinants of the defaulter to full vaccination at a community level is important to address and overcome the reasons for not having full vaccination. Therefore, this study aimed to identify determinants of defaulter to full vaccination among children aged 12 to 23 months in Siraro District, West Arsi Zone, Ethiopia.

## Methods and materials

### Study Design, Period and Area

A Community-based unmatched case–control study design was conducted from March 20 to April 30, 2022 at Siraro District. The district is found in the West Arsi Zone and is composed of 4 urban and 28 rural kebeles. The Kebele is the smallest administrative unit below the district according to the Ethiopian context. The District is located in Southwest Ethiopia at 64 Km far from the capital city of zone, Shashemene and 314 Km from the capital city of the country, Addis Ababa. In line with the national immunization strategies, the district is providing Expanded Program on Immunization services through static and outreach strategies. The district has 38 outreach sites providing immunization services for communities living around more than 5 km from the nearest health facility. The District Health Office is directly involved in providing support to primary health care units through conducting supportive supervision and quarterly review meetings.

According to the Siraro District Health Office estimate in 2021/22, the total population in the District is 217,572 with 35,749 under-five children and an estimated number of children aged 12–23 months were calculated using converting factor of 45.67% from under five children (35,749 × 45.67/100 = 16,326). The District has 1 Primary Hospital, 7 Health Centers, and 27 functional Health Posts. There are also 139 health professionals and 54 health extension workers (HEWs) in the district (Source: Siraro District Health Office).

### Source Population and Study Population

The study population were all children aged 12 to 23 months with their mothers/ caretakers who had received at least one dose of the routine vaccination and living in eligible households from randomly selected kebeles in Siraro District. The cases were children between the ages of 12–23 months with their mothers/ caretakers who had missed at least one dose of the recommended routine vaccination for any reason and were not fully vaccinated for age as per the national immunization schedule. The controls were children aged 12–23 months with their mothers /caretakers who had received all the recommended routine vaccination as per the national immunization schedule for full vaccination by the age of their first birth year.

### Eligibility criteria

Children aged 12–23 months with their mothers/caretakers living in Siraro District for at least 6 months before the day of data collection. Children who had a full address from the Family folder and lived with their mothers/ caretakers at the time of the study and who are permanent residents in Siraro District were included in this study. Child mothers/caretakers who had missed child vaccination cards and have no date of childbirth (whose vaccination cards were not available or lost were excluded to reduce recall and selection bias) and those unable to interview due to illness and hearing problems.

### Sample size determination

The sample size was calculated based on the double population proportion formula by using Epi Info statistical software version 7.2 for unmatched case–control study design by considering the determinant variables from previous case-control studies conducted in Ethiopia and compared to get a large sample[5, 13]. The predictor variable not attended postnatal care follow up considered a significant determinant of the defaulter to full vaccination from previous studies since it gave the maximum sample size. Among controls, 45.9% of mothers not attended postnatal care follow-up, while among cases it was 75.3% and OR = 3.58 [5]. Using the assumptions of 80% power (Z*β =* 0.84), 95% confidence level (Z*α*/2 = 1.96), a case-to-control ratio of 1:2, and after adding a 10% non-response rate and design effect of 1.5, the total sample size was 444 (148 cases and 296 controls).

#### Sampling procedures

**Fig. 1:**
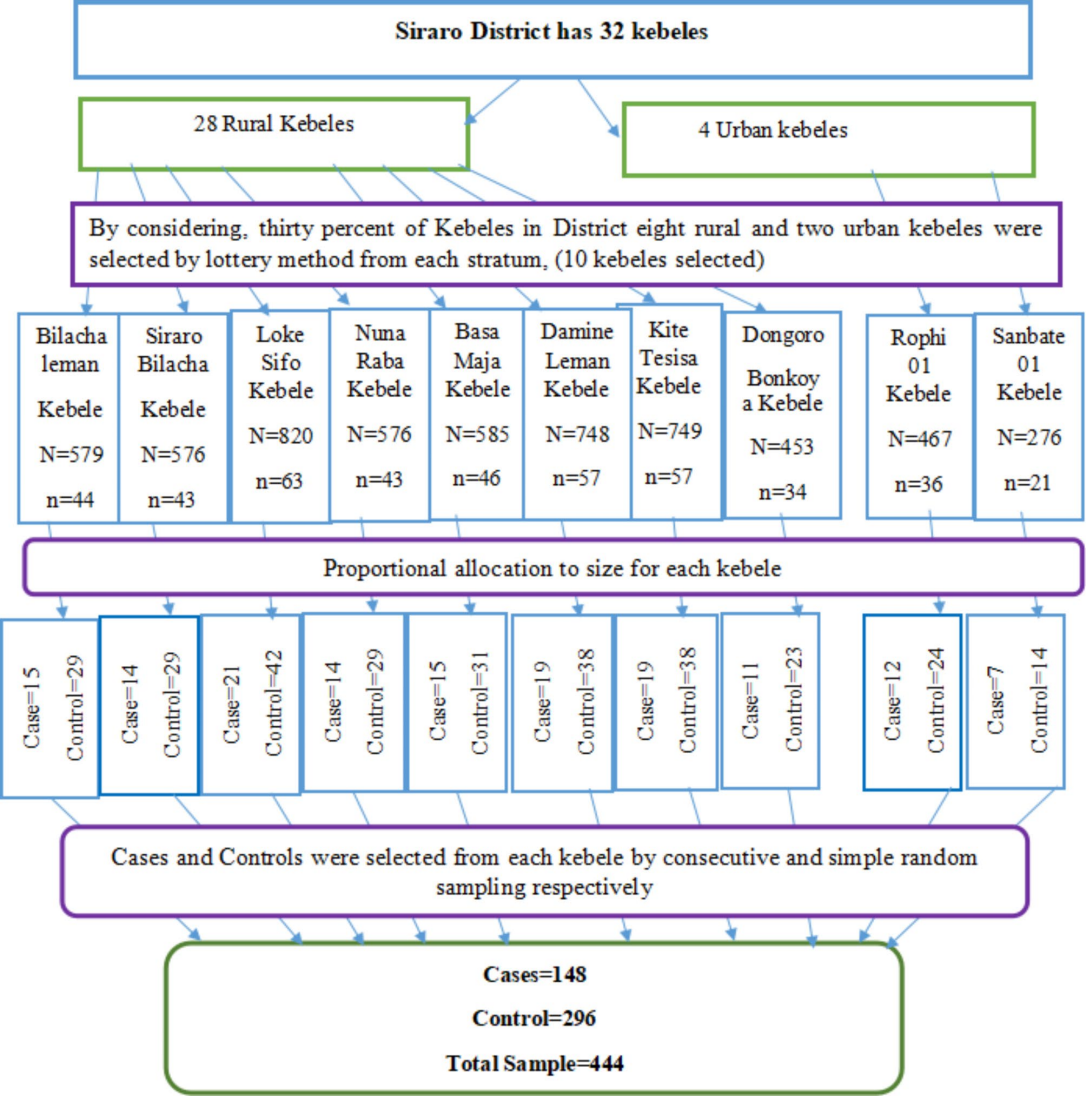
Schematic representation of sampling procedures on determinants of the defaulter to full vaccination among children aged 12–23 months in Siraro District, 2022

The appropriate representative kebeles and households were selected by using multistage sampling from 32 kebeles in the district. Initially, the kebeles found in the district were stratified into urban and rural. There are 28 rural and 4 urban kebeles in the district. Eight rural and two urban kebeles were selected randomly using the lottery method by considering the rule of thumb (30%) of kebeles from each stratum (Fig. 1). All eligible cases and controls were listed with their full address from the Family folder of nearby health posts separately for each selected kebeles of rural and urban before actual data collection. A family folder is a registry book containing all family members’ profiles in the kebele.

Finally,148 cases were selected using consecutive sampling based on the principles of subsequent nearest eligible households and 296 controls were selected by using a simple random sampling technique after proportional allocation to the number of children aged 12 to 23 months to the number of children in each kebele using a computer-generated technique. When there are two or more children in an eligible household one of them were selected using the lottery method. In case of absenteeism of respondents during the visit, after checking three times visits the next eligible households were included.

### Study variables

#### Dependent variable

Defaulter to full vaccination status

#### Independent variables

**Sociodemographic characteristics of mother/caretakers** (age, marital status, educational status occupation, residence, family size, income)

**Child biographic-related characteristics** (child age, sex, birth order, birth interval)

**Vaccination service delivery-related characteristics** (time to reach a health facility, waiting time, postponing schedule, parents’ discussion on vaccination)

**Knowledge, Attitude, and Service Satisfaction** of mothers/ caretakers on child vaccination

**Maternal Health service utilization-related characteristics** (Antenatal care visit, postnatal care visit, place of delivery, pregnant mother conference participation, model family training)

### Measurements and operational definitions

**Defaulter (cases)**: A child aged 12 to 23 months old who had started the routine immunization and missed at least one dose of the recommended routine vaccination (BCG, DPT-HepB -Hib, Polio vaccine, Pneumococcal conjugate vaccine (PCV), IPV and Measles vaccine) schedule for any reason was considered as a defaulter [8]. **Full vaccination (controls)**: A child aged 12–23 months who had received all the recommended routine vaccination as per the national immunization schedule in Ethiopia (BCG, DPT-HepB -Hib, Polio vaccine, Pneumococcal conjugate vaccine (PCV), IPV and Measles vaccine) for full vaccination by the age of their first birth year were considered as full vaccination [8].

#### Knowledge about child vaccination

Mothers/caretakers were asked ten questions which are related to child vaccination using “Yes, No and I don’t know” options. The correct answers were given a value of 1 and 0 for incorrect answers. After computing, the sum score for each respondent, the minimum points to be scored were 0 and the maximum 10. The mean value of the knowledge score (6.22) was used as a cut-off point to categorize knowledge into two groups. The variable was dichotomized into having “adequate knowledge” about vaccination for those respondents who scored greater than or equal to the mean score of knowledge points (≥ 6.22) and “inadequate knowledge” about vaccination for those respondents who scored less than the mean score of knowledge points (< 6.22) [4, 13].

#### Attitude toward child vaccination

Six questions were used to assess mothers’/caretakers’ attitude towards child vaccination using five-point Likert scale questions that have 1 to 5 options, were 1 = strongly disagree, 2 = disagree, 3 = neutral, 4 = agree, and 5 = strongly agree. The sum score of mothers’/caretakers’ attitude were computed and converted to give the minimum and maximum points of caretakers’ attitude score from 6 to 30 respectively. The variable was dichotomized into having a “favorable attitude” toward child vaccination if the mothers/caretakers’ attitude score value greater than the mean score (> 16.57), and “unfavorable attitude” toward child vaccination if the mothers/caretakers’ attitude score value less than or equal to mean score (≤ 16.57) [14].

#### Satisfaction towards child vaccination

Mothers/caretakers were asked seven service satisfaction-related questions using a five-point Likert scale questions that starts from 1 to 5 options (were 1 = strongly disagree, 2 = disagree, 3 = neutral, 4 = agree, and 5 = strongly agree). Each variable was measured on 5 points and which together yield a minimum of 7 and a maximum of 35. A response to 7 measuring items was added and converted to give an individual level of mothers/caretakers satisfaction score from 1 to 100% for each item. The variable was dichotomized into “satisfied” and “not satisfied”. Mothers/caretakers who scored 75% and above on 7 satisfaction measuring items were considered as “satisfied”, and those who scored less than 75% as “not satisfied” [5].

#### Caretaker

Caretaker is the most responsible person who provides care to the child other than the mother due to different reasons such as the parents’ death, adoption of a child, separation from family, and others [8, 17].

### Data Collection Tool and procedures

Data were collected using an interviewer-administered structured questionnaire to obtain information from mothers/caregivers of the child by trained interviewers. The instrument was constructed from a review of available literature on child immunization [4, 5, 13, 14]. The questionnaire has seven parts, which include sociodemographic characteristics of respondents, child biographic characteristics, child vaccination service delivery characteristics, mothers/caretakers’ knowledge of child vaccination, mothers/caretakers’ attitude towards child vaccination, immunization service satisfaction, and maternal health service utilization characteristics.

The mother’s/caregivers’ knowledge and attitude were assessed by ten and six questions respectively. Service satisfaction was assessed by seven questions related to immunization service satisfaction. For the assessment of mothers/caretakers’ attitude and service satisfaction five-point Likert scale questions were used and each questionnaire measures Likert scale response options scored from 1 to 5, (were 1 = strongly disagree, 2 = disagree, 3 = neutral, 4 = agree, and 5 = strongly agree).

Six Diploma Nurses as data collectors and three BSc health professional supervisors were recruited based on a set of criteria, which includes the understanding of the local language, Afan Oromo, and previous data collection experience. Data Collectors were trained for two days by the investigator on the purpose of the study, data collection tools or instruments, how to take consent, how to select children from households, how to interview and extract information from vaccination cards, and the overall data collection procedures. If there are two or more children available in an eligible household one of them were selected using the lottery method.

### Data Quality Assurance

The questionnaire was prepared in English, translated into Afaan Oromo (the local language of the area), and retranslated to English by another translator who is a health professional to ensure the consistency of the tool. A Pretest of the questionnaire was done on mothers/caregivers by taking 5% of the total sample size (23 participants with 8 cases and 15 controls) in two kebeles that were not included in the study before actual data collection to identify any problems and check the clarity by ensuring the tool accurately addressed the research questions.

After the pretest, the investigator, data collectors, and supervisors were discussed on the questionnaire for any inconsistencies and ambiguity before actual data collection and the necessary adjustments were made to the questionnaire before use for actual data collection. The content validity of the tool was cross-checked by another health professional. Data collectors and supervisors were trained on the study instrument and data collection procedure. The investigator and supervisors checked the collected data for consistency and completeness every day at the end of each data collection day and necessary corrective measures were taken accordingly.

### Data processing and analysis

The collected data were checked for completeness, consistency and accuracy. Data were coded and entered into Epi data manager version 4.6, cleaned, and analyzed using SPSS version 26.0. After cleaning data for inconsistencies and missing values, descriptive statistics were used to present the data with the different variables by tables and graphs. The binary logistic regression was used to identify the determinant variables. To control potential confounders, independent variables with P-value < 0.25 in the bivariable analysis were considered for multivariable logistic regression analysis [4, 5, 14], and the multi-collinearity of independent variables was checked by using Standard error (SE). The Standard error of 2 was used as a cut-off point to indicate the presence of multi-collinearity between independent variables however; in this study, it is found to be less than 2 standard error.

The model fitness test was checked by Hosmer and Lemeshow’s goodness of test and the model was fit to data. The statistical significance of the variables were interpreted using an adjusted odd ratio (AOR) with a 95% confidence interval and p-value ≤ 0.05 to identify independent determinants of the defaulter to full vaccination. Finally, the result was presented in the form of text, using tables, figures, and charts.

## Result

### Sociodemographic characteristics of study participants

A total of 437 study participants (145 cases and 292 controls) were included in this study and making a response rate of 98.4%. The mean age of study participants were 28.42 (SD = ± 5.83) years for cases and 27.10 (SD = ± 5.69) years for controls, which ranges from 17 to 40 years for cases and 17–42 years for controls. The majority of child mothers/caretakers’ marital status and Religion were married and Muslim, 138 (95.2%) and 122 (84.1%) among cases and 275 (94.2%) and 222 (76%) among controls respectively.

Concerning the educational status of study participants, 101 (69.7%) of cases and 163 (55.8%) of controls were unable to read and write. Regarding the occupational status of child mothers/caretakers 117 (80.7%) of cases and 231 (79.1%) of controls were housewife. Forty-one (28.3%) of cases and 77 (26.4%) of the controls had a family size greater than or equal to five. Sixty-eight (51.9%) households of cases and 134 (51.1%) of controls had an average household monthly income of less than or equal to 1000 Ethiopian Birr (Table 1).

**Table 1:** Socio-demographic characteristics of child mothers/caretakers on determinants of defaulter to full vaccination among children aged from 12 to 23 months in Siraro District, West Arsi Zone, Oromia, Ethiopia 2022 (n = 437)

Variables	Cases, n (%)	Control, n (%)
**Age of respondents (in a year)**		
≤ 24	42 (29.0)	109 (37.3)
25–29	38 (26.2)	93 (31.9)
30–34	38 (26.2)	48 (16.4)
≥ 35	27 (18.6)	42 (14.4)
**Marital status**		
Married	138 (95.2)	275 (94.2)
Others marital status^**a**^	7 (4.8)	17(5.8)
**Religion**		
Muslim	122 (84.1)	222 (76.0)
Protestant	13 (9.0(	41 (14.0)
Orthodox	10 (6.9)	29 (10.0)
**Residence**		
Rural	126 (86.9)	254 (87.0)
Urban	19 (13.1)	38 (13.0)
**Education of Mother**		
Unable to read and write	101 (69.7)	163 (55.8)
Primary (1–8)	27 (18.6)	86 (29.5)
Secondary (9–12) and above	17 (11.7)	43 (14.7)
**Education of Father**		
Unable to read and write	93 (64.1)	148 (50.7)
Primary (1–8)	40 (27.6)	92 (31.5)
Secondary (9–12) and above	12 (8.3)	52 (17.8)
**Occupation of Mother**		
Housewife	117 (80.7)	231 (79.1)
Merchant	15 (10.3)	41 (14.0)
Private employee	7 (4.8)	5 (1.8)
Government employee	6 (4.1)	15 (5.1)
**Occupation of Father**		
Farmer	109 (75.2)	208 (71.2)
Merchant	23 (15.9)	60 (20.5)
Private employee	7 (4.8)	6 (2.1)
Government employee	6 (4.1)	18 (6.2)
**Family size**		
Less than five	104 (71.7)	215 (73.6)
Greater than or equal to five	41 (28.3)	77 (26.4)
**Monthly income (ETB)**		
≤ 1000	68 (51.9)	134 (51.2)
1001–2000	29 (22.1)	49 (18.7)
2001–3000	14 (10.7)	16 (6.1)
≥ 3001	20 (15.3)	63 (24.0)

^**a**^ **=** *marital status of respondents including widowed, divorced, single*

### Child biographic-related characteristics

The mean age of children was 16.77 (SD = ± 3.09) months among cases and 17.21 (SD = ± 3.11) months among controls. About half of the children under the study were male by sex, 71 (49.0%) among cases and 148 (50.7%) among controls. Concerning birth order of the child 66 (45.5%) among cases and 105 (36%) among controls were born fourth and above birth order. The median birth interval of children under the study was 24.0 (IQR = 6.33) months among cases and 24.0 (IQR = 6.35) months among controls respectively. The majority of respondents relation to the child were mothers, 125 (86.2%) among cases and 255 (87.3%) among controls (Table 2).

**Table 2:** Child biographic-related characteristics on determinants of defaulter to full vaccination among children aged from 12 to 23 months in Siraro District, West Arsi Zone, Oromia, Ethiopia 2022 (n = 437)

Variables	Cases, n (%)	Control, n (%)
**Sex of the child**		
Male	71 (49.0)	148 (50.7)
Female	74 (51.0)	144 (49.3)
**Birth order of the child**		
First birth order	20 (13.8)	40 (13.7)
Second birth order	30 (20.7)	88 (30.1)
Third birth order	29 (20.0)	59 (20.2)
Fourth and above birth order	66 (45.5)	105 (36.0)
**Birth Interval (in months)**		
< 24	67 (53.6)	145 (57.5)
24–36	53 (42.4)	93 (36.9)
> 36	5 (3.4)	14 (5.6)
**Respondents’ relation to child**		
Mother	125 (86.2)	255 (87.3)
Caretaker	20 (13.8)	37 (12.7)

### Knowledge, attitude and service satisfaction-related characteristics

**Fig. 2:**
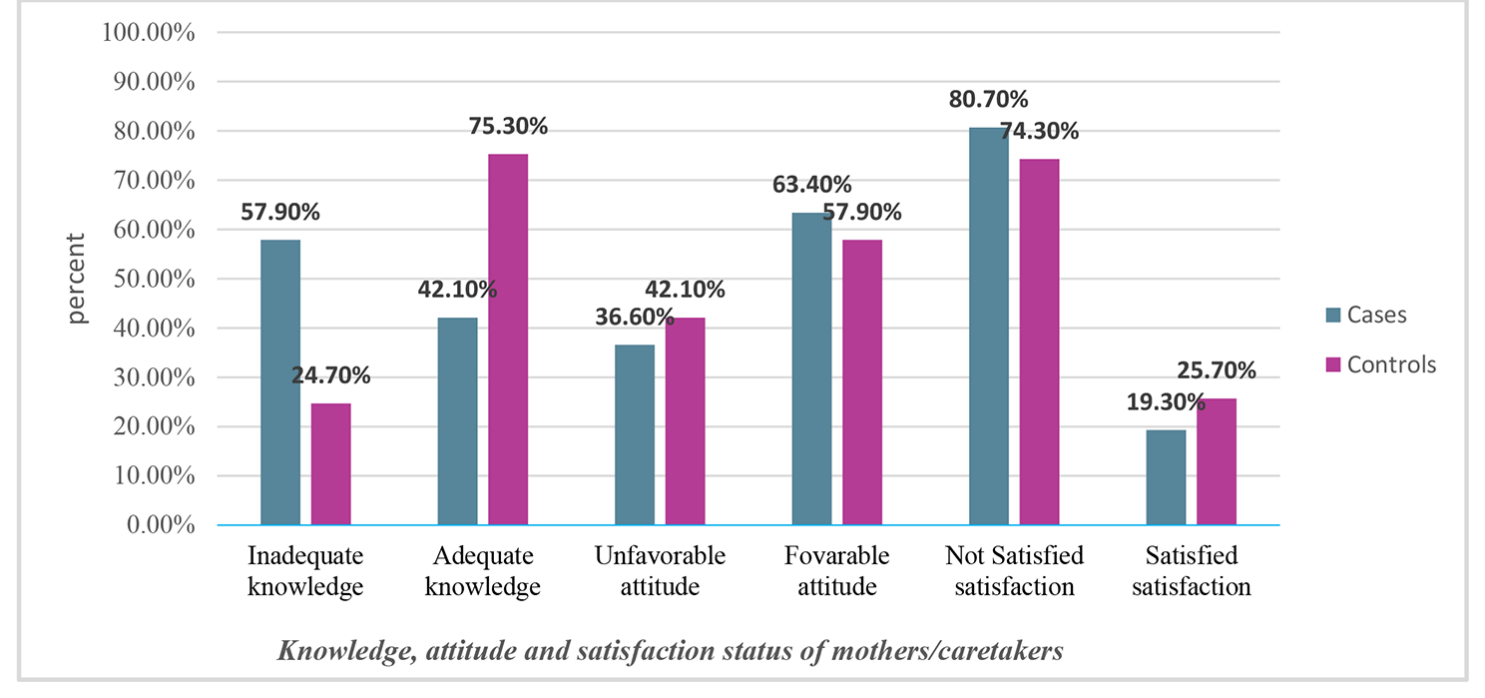
Knowledge, Attitude, and Service Satisfaction status of child mothers/caretakers on determinants of the defaulter to full vaccination among children aged 12 to 23 months in Siraro District, West Arsi Zone, Oromia, Ethiopia 2022

Of the interviewed child mothers/caretakers 84 (57.9%) among cases and 72 (24.7%) among controls had inadequate knowledge about child vaccination. Concerning mothers’/caretakers’ attitude towards child vaccination 53 (36.6%) among cases and 23 (42.1%) among controls had an unfavorable attitude towards child vaccination. Regarding vaccination service satisfaction by child mothers/caretakers 117 (80.7%) cases and 217 (74.3%) among controls were not satisfied with child vaccination service (Fig. 2).

### Child Vaccination Service Delivery-related characteristics

Concerning time to reach the health facilities for child vaccination by mothers/caretakers, 74 (51.0%) cases and 86 (29.5%) among controls had spent greater than 30 min to reach the vaccination site on a foot walk time. About 43 (29.7%) mothers/caretakers of cases and 51 (17.5%) of controls had spent greater than or equal to fifteen minutes for waiting time at a health facility during child vaccination. About 78 (53.8%) mothers/caretakers of cases and 124 (42.5%) controls had no access to media.

**Fig. 3:**
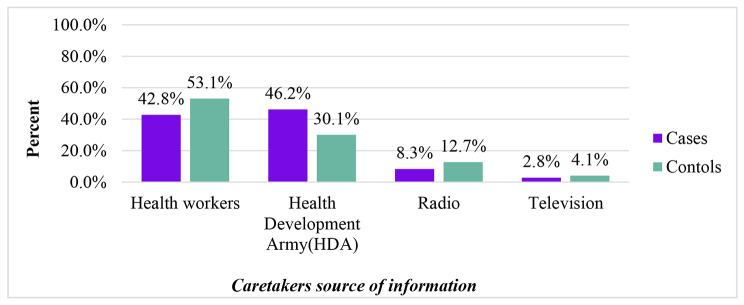
Mothers/Caretakers’ source of information on determinants of defaulters to full vaccination among children aged 12–23 months in Siraro District, West Arsi Zone, Oromia, Ethiopia 2022

Concerning mothers/caretakers’ information about vaccination 54 (37.2%) mothers of cases and 46 (15.8%) mothers of controls didn’t get information about possible vaccine side effects and 94 (64.8%) mothers/caretakers of cases and 267 (91.4%) of controls had got information about next vaccination schedules. About 88 (60.7%) mothers/caretakers of cases and 211 (72.3%) controls were faced with postponing the vaccine schedules during they visited a health facility for child vaccination (Table 3). Concerning child mothers/caretakers’ source of information for child vaccination 62 (42.8%) of cases and 155 (53.1%) of controls were got information from Health workers (Fig. 3).

**Table 3:** Child Vaccination Service Delivery related characteristics on determinants of defaulter to full vaccination among children aged from 12 to 23 months in Siraro District, West Arsi Zone, Oromia, Ethiopia 2022 (n = 437)

Variables	Cases, n (%)	Control,n (%
**Time to reach health facility (in walk time)**		
Less than or equal to 30 min	71 (49.0)	206 (70.5)
Greater than 30 min	74 (51.0)	86 (29.5)
**Waiting time at health facility (minutes)**		
Less than 15 min	102 (70.3)	241 (82.5)
Greater than or equal to 15 min	43 (29.7)	51 (17.5)
**Means of transportation to health Facility**		
On foot walk	98 (67.6)	196 (67.1)
By transportation	47 (32.4)	96 (32.9)
**Does transportation means incur payments**		
Yes	41 (87.2)	81 (84.4)
No	6 (12.8)	15 (15.6)
**Access to media**		
Yes	67 (46.2)	168 (57.5)
No	78 (53.8)	124 (42.5)
**Counseled on the benefit of immunization by HEWs**		
Yes	105 (72.4)	269 (92.1)
No	40 (27.6)	23 (7.9)
**Health workers told the type of vaccine received**		
Yes	74 (51.0)	236 (80.8)
No	71 (49.0)	56 (19.2)
**Information about possible vaccine side effects**		
Yes	91 (62.8)	246 (84.2)
No	54 (37.2)	46 (15.8)
**Information on next vaccine schedule**		
Yes	94 (64.8)	267 (91.4)
No	51 (35.2)	25 (8.6)
**Postponing vaccine schedule**		
Yes	88 (60.7)	211 (72.3)
No	57 (39.3)	81 (27.7)
**Parents’ discussion on immunization**		
Yes	67 (46.2)	215 (73.6)
No	78 (53.8)	77 (26.4)

### Maternal Health Service utilization related characteristics

Of the child mothers, 103 (71.0%) among cases and 263 (90.1%) among controls attended ANC follow-up. Among those mothers who had attended ANC follow-up, only 26 (25.2%) of cases and 84 (31.9%) of controls were attended the fourth and above visit. Concerning child mothers’ participation in pregnant mother conferences 94 (64.8%) among cases and 258 (88.4%) among controls have participated in pregnant mothers conferences. Of these mothers, only 67 (46.2%) among cases and 191 (65.4%) among controls were counseled on child vaccination after delivery. Forty-four (30.3%) mothers of cases and 176 (60.3%) controls attended PNC follow-up. Of the interviewed child mothers, 34 (23.4%) among cases and 134 (45.9%) among controls attended model family training. About 71 (49.0%) mothers of cases and 234 (80.1%) controls gave birth at a health facility (Table 4).

**Table 4:** Maternal Health Service Utilization related characteristics on determinants of defaulter to full vaccination among children aged from 12 to 23 months in Siraro District, West Arsi Zone, Oromia, Ethiopia 2022 (n = 437)

Variables	Cases, n (%)	Control, n (%)
**Attended antenatal care follow up**		
Yes	103 (71.0)	263 (90.1)
No	42 (29.0)	29 (9.9)
**Number of ANC Visit attended**		
First Visit	13 (12.6)	33 (12.5)
Second Visit	33 (32.0)	71 (27.1)
Third Visit	31 (30.1)	75 (28.5)
Fourth and above Visit	26 (25.3)	84 (31.9)
**Tetanus Toxoid Vaccination during ANC Visit**		
Yes	89 (86.6)	241 (91.6)
No	14 (13.4)	22 (8.4)
**Participation in Pregnant Mother Conference**		
Yes	94 (64.8)	258 (88.4)
No	51 (35.2)	34 (11.6)
**Place of delivery**		
Health facility	71 (49.0)	234 (80.1)
Home	74 (51.0)	58 (19.9)
**Counseled on child immunization after delivery**		
Yes	67 (46.2)	191 (65.4)
No	78 (53.8)	101 (34.6)
**Attended PNC follow up**		
Yes	44 (30.3)	176 (60.3)
No	101 (69.7)	116 (39.7)
**Number of PNC Visits**		
Only one visit	32 (72.7)	98 (55.7)
Two and above visit	12 (27.3)	78 (44.3)
**Using a modern contraceptive method**		
Yes	59 (40.7)	187 (64.0)
No	86 (59.3)	105 (36.0)
**Attended model family training**		
Yes	34 (23.4)	134 (45.9)
No	111 (76.6)	158 (54.1)
**Graduated as a model family**		
Yes	19 (13.1)	102 (34.9)
No	126 (86.9)	190 (65.1)

### The most defaulted vaccine types by children

**Fig. 4:**
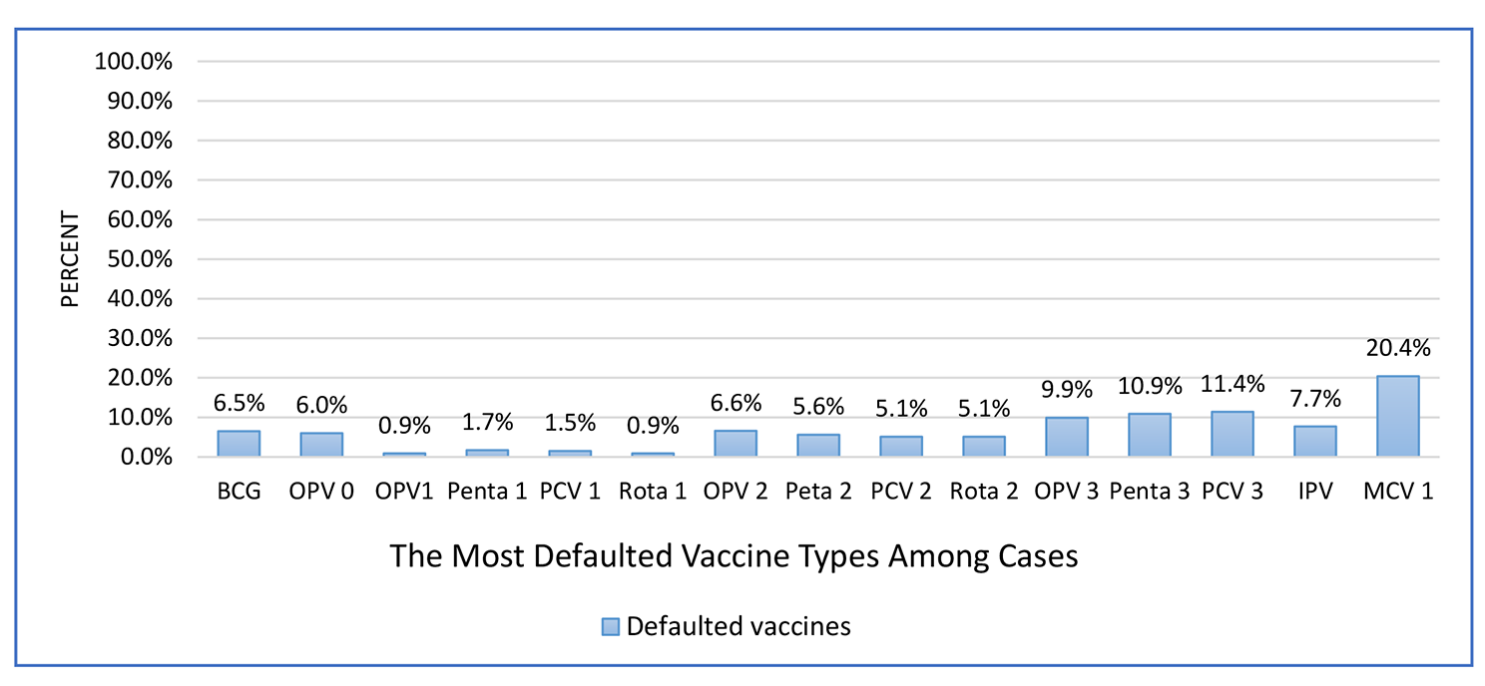
The most defaulted vaccine types by children aged 12–23 months in Siraro District, West Arsi Zone, Oromia, Ethiopia 2022

Measles (20.4%), PCV3 (11.4%), and Penta3 (10.9%) were the most defaulted vaccines types that were identified from selected child mothers/caretakers’ history with cross-checking from children’s vaccination cards (Fig. 4).

### Determinants of defaulter to full vaccination

Of all assessed determinants of the defaulter to full vaccination fourteen variables show association in bivariable analysis; knowledge of mothers/caretakers, educational status of the mother, educational status of the father, average monthly income, time to reach a health facility, health workers told types of vaccine received, next vaccine schedule, service satisfaction, ANC follow up, place of delivery, PNC follow up, attended model family training, participation in pregnant mother conference and parents discussion on immunization.

All variables with a p-value < 0.25 in the bivariable analysis were entered into multivariable logistic regression analysis after adjusting for confounders. The odds of the defaulter to full vaccination of the children were 4.32 times more likely higher among child mothers/caretakers who had inadequate knowledge about child vaccination as compared to those who had adequate knowledge of child vaccination (AOR = 4.32, 95% CI:2.79–6.70).

The odds of the defaulter to full vaccinations of the children were 3.6 times more likely among unable to read and write educational status of child fathers as compared to those who attended secondary [9–12] and above education (AOR = 3.66, 95% CI:1.29–10.39). The child born from mothers /caretakers who had spent time taken to reach a health facility greater than 30 min were 2.45 times higher odds of the defaulter to full vaccination than those mothers/caretakers who spent less than or equal to 30 min (AOR = 2.45, 95% CI:1.51–3.97). The result of this study signifies that the odds of the defaulter to full vaccination of children were 2.37 times more likely higher among child mothers/caretakers who didn’t told about the type of vaccine received by health workers than those who had been told the type of vaccine received (AOR = 2.37, 95% CI:1.27–4.45).

The odds of the defaulter to full vaccination of the children were 2.16 times more likely higher among child mothers/caretakers who had no parents discussion on child immunization as compared to their counterparts (AOR = 2.16, 95% CI:1.24–3.79). This study also showed that child mothers/caretakers who had given the birth at home were 2.43 times more likely higher odds of the defaulter to full vaccination of their children as compared to those who had given the birth at a health facility (AOR = 2.43, 95% CI:1.39–4.25). The odds of the defaulter to full vaccinations of the children were 2.47 times higher among child mothers/caretakers who did not participated in the pregnant mother conference than those who had participated in the pregnant mother conference (AOR = 2.47, 95% CI:1.35–4.49) (Table 5).

**Table 5:** Bivariable and Multivariable analysis on determinants of the defaulter to full vaccination among Children Aged 12–23 Months in Siraro District, West Arsi Zone, Ethiopia 2022 (n = 437)

Variables	Cases, n(%)	Control, n(%)	COR(95%CI)	AOR(95%CI)	P Value
**Knowledge about child vaccination**					
Inadequate knowledge	84 (57.9)	72 (24.7)	4.20 (0.2.75, 6.43)*	**4.32 (2.79, 6.70)**	< 0.001**
Adequate knowledge	61 (42.1)	220 (75.3)	1	**1**	
**Educational status of Mothers/caretakers**					
Unable to read and write	101 (69.7)	163 (55.8)	1.57 (0.85, 2.89)*	1.01 (0.44, 2.33)	0.97
Primary (1–8)	27 (18.6)	86 (29.5)	0.79 (0.39, 1.61)	0.60 (0.25, 1.43)	0.25
Secondary (9–12) and above	17 (11.7)	43 (14.7)	1	**1**	
**Educational status of Fathers**					
Unable to read and write	93 (64.1)	148 (50.7)	2.72 (1.38, 5.37)*	**3.66 (1.29, 10.39)**	0.02**
Primary (1–8)	40 (27.6)	92 (31.5)	1.88 (0.91, 3.91)	2.42 (0.88, 6.61)	0.85
Secondary (9–12) and above	12 (8.3)	52 (17.8)	1	1	
**Average monthly income (ETB)**					
≤ 1000	68 (51.9)	134 (51.1)	1.59 (0.89, 2.86) *	1.20 (0.48, 3.03)	0.69
1001–2000	29 (22.1)	49 (18.7)	1.86 (0.94, 3.68)	1.42 (0.56, 3.61)	0.45
2001–3000	14 (10.7)	16 (6.1)	2.75 (1.15, 6.62)	1.43 (0.73, 2.74)	0.13
≥ 3001	20 (15.3)	63 (24.0)	1	1	
**Time to reach health facility (in walk time)**					
Less than or equal to 30 min	71 (49.0)	206 (70.5)	1	1	
Greater than 30 min	74 (51.0)	86 (29.5)	2.49 (1.65, 3.76)*	**2.45 (1.51, 3.97)**	< 0.001**
**Health workers told type of vaccine received**					
Yes	74 (51.0)	236 (80.8)	1	1	
No	71 (49.0)	56 (19.2)	4.04 (2.61, 6.25)*	**2.37 (1.27, 4.45)**	0.01**
**Information about next vaccine schedul**e					
Yes	94 (64.8)	267 (91.4)	1	1	
No	51 (35.2)	25 (8.6)	5.79 (3.40, 9.87)*	2.28 (0.94, 5.55)	0.71
**Parents’ discussion on child immunization**					
Yes	67 (46.2)	215 (73.6)	1	1	
No	78 (53.8)	77 (26.4)	3.25 (2.14, 4.93)*	**2.16 (1.24, 3.79)**	0.01**
**Immunization Service Satisfaction**					
Not satisfied	117 (80.7)	217 (74.3)	1.44 (0.89, 2.36)*	1.46 (0.87, 2.44)	0.152
Satisfied	28 (19.3)	75 (25.7)	1	**1**	
**Attended ANC follow up**					
Yes	103 (71.0)	263 (90.1)	1	**1**	
No	42 (29.0)	29 (9.9)	3.69 (2.19, 6.25)*	2.70 (0.95, 2.13)	0.62
**Place of delivery**					
Health facility	71 (49.0)	234 (80.1)	1	**1**	
Home	74 (51.0)	58 (19.9)	4.21 (2.72, 6.49)*	**2.43 (1.39, 4.25)**	0.002**
**Attended PNC follow up**					
Yes	44 (30.3)	176 (60.3)	1	**1**	
No	101 (69.7)	116 (39.7)	3.48 (2.28, 5.32)*	1.64 (0.95, 2.81)	0.074
**Participation in pregnant mother conference**					
Yes	94 (64.8)	258 (88.4)	1	**1**	
No	51 (35.2)	34 (11.6)	4.12 (1.44, 3.26)*	**2.47 (1.35, 4.49)**	0.003**
**Attended model family training**					
Yes	34 (23.4)	134 (45.9)	1	**1**	
No	111 (76.6)	158 (54.1)	2.76 (1.76, 4.33)*	1.16 (0.58, 2.33)	0.63

AOR = Adjusted Odds Ratio, COR = Crude Odds Ratio, CI = Confidence Interval, (*) Variable category with p value < 0.25 in bivariable analysis, (**) statistically significant at p-value < 0.05, **1 =** Reference group

## Discussion

This study aimed to assess determinants of defaulter to full vaccination among children aged 12–23 months in Siraro District, Ethiopia. In this study, the identified determinants of the defaulter to full vaccination were inadequate knowledge of mothers/caretakers, unable to read and write the educational status of child fathers, time to reach health facility greater than 30 min, home delivery, health workers who were not being told the type of vaccine received, caretakers who did not participate in pregnant mother conference and no parents discussion on child immunization.

Knowledge of mothers/caretakers about child vaccination was associated with defaulter to full vaccination. Children born from mothers /caretakers who had inadequate knowledge of child vaccination were more likely to default from full vaccination than those who had adequate knowledge of child vaccination.This finding is consistent with a study done in Gindhir district, Wadla district, Nigeria, and Rwanda [11, 13, 20, 21]. This indicates that, poor knowledge of mothers on child immunization can lead to defaulters to full vaccination. This might be due to child mothers who don’t know more about childhood vaccination and those not getting awareness about immunization may be reluctant in completing their child’s vaccination based on appropriate vaccine schedules. It is well recognized that knowledge of caretakers towards child vaccination plays an important role in mothers utilization of vaccination service and help them to have an interest to vaccinate their child.

According to this study, the educational status of fathers was another determinant that showed a significant association with defaulters to the full vaccination status of children. Those children born from fathers unable to read and write educational status were positively associated with defaulter to full vaccination than those fathers who attended secondary and above education. This result is consistent with the study conducted in the Pawi district [22], Sinana district [23], and Nigeria [21]. It indicates that, child fathers who had no formal education could have inadequate information about vaccine-preventable diseases and the use of child vaccination. This might be due to those uneducated fathers could have no adequate understanding and decision-making skills on different child health service including the importance of child vaccination. If the fathers of the children have rgular education and sufficient information on child vaccination, they can encourage the child mothers for vaccination of their child as per routine immunization schedules.

The time taken to reach the health facilities was found to be one of the independent determinants of the defaulter to full vaccination. Child mothers/caretakers who had spent greater than 30 min to reach the health facility were more likely to default their child from full vaccination than those who had spent less than or equal to 30 min. This finding is in line with a study done in the Laelay Adiabo District of Tigray region and Debra Markos Town [4, 24]. Studies conducted in Kenya and Somalia Putland also found that the long time to the vaccination site was a determinant of the defaulter to full vaccination [25, 26]. This could be due to the long time spent to reach health facilities for child vaccination costs to the mothers/caretakers and it may have the effect on the child mothers/caretakers by dissatisfying of them due to long time to reach health facilities and results for defaulting of their children from completion of the routine immunization.

The result of this study revealed that child mothers/caretakers who did not told about the type of vaccine received by health workers were two times more likely to have defaulter to full vaccination of their children than those who had been told the type of vaccine received by health workers. The study conducted at Woldia Town in Ethiopia supports this finding. Which indicates that lack of awareness about the type of vaccine received was the factor for defaulters to full vaccination of children [27]. This might be due to mothers/caretakers may forgot whether the child received all the types of recommended vaccines. In addition, health care providers may not properly counsel on the next appointments and types of vaccine given to the child which may lead to the defaulter to full vaccination of the children. Telling key messages of the routine immunization to the caretakers plays an important role to understand the types of vaccine received by children and helps to complete all the recommended vaccines without defaulting from the full vaccination.

The study also found that mothers/caretakers who had no parents ever discussion on child immunization were 2 times more likely to have defaulter to full vaccination of their children than those who had a parental discussion on child vaccination. This finding is consistent with a study done in Sinana District [23] and Afghanistan [28]. It shows that the likelihood of defaulting from full vaccination of the child immunization was higher among mothers who have poor parents’ discussions on child vaccination. This could be due to the household decision-making power of the father and the awareness on Vaccine Preventable Diseases may influences child mothers/caretakers to vaccinate their children. Those child parents who have regular discussion on vaccination can have a continiuos attention to vaccinate their children.

A child born from mothers/caretakers who had gave birth at home were positively associated with defaulter to full vaccinations than those who had gave birth at the health facility. The finding is consistent with the study conducted in different parts of Ethiopia [8, 22, 29] and Somalia [30]. It indicates that giving birth at home had a direct and significant association with defaulter to the full vaccination status of the children. This could be due to child mothers/caretakers who had gave birth at home can miss the chance of getting information on vaccination and health education about child immunization. This may cause the mothers/caretakers to default their children from completing full vaccination.

The child mothers/caretakers who did not participated in the pregnant mother conference had more likelihood of defaulter to full vaccinations of their children than those who had participated in the pregnant mother conference. The finding is in line with a study done in Afar region [31] and Oromia region in Central Ethiopia [29]. This might be due to the mothers/caretakers who had not attended pregnant women conferences lacking more information and understanding on child vaccination, not having sufficient awareness about vaccine-preventable diseases, and not knowing more about the importance of vaccines. This could may enhances the risk of the defaulter to full vaccination of the children.

### Strength and limitation of the study

Being a community-based study and excluding those child mothers/caretakers who had missed child vaccination cards and vaccination cards had no date of childbirth to reduce recall and selection bias gives the strength for this study. As limitations, the risk factors of defaulters of vaccination may differ among the child mothers/caretakers need investigation through detailed follow-up of the defaulters’ processes from basic roots that require prospective study was not applied in this study.

## Conclusion

The finding of this study were assessed and identified different factors for defaulter to full vaccination. Of all assessed determinants of defaulters of the childhood vaccination; inadequate knowledge of mothers/caretakers’ about child vaccination, unable to read and write the educational status of child fathers, time to reach health facility greater than 30 min, child mothers/caretakers who didn’t told about the type of vaccine received by health workers, home delivery, no parents discussion on child vaccination and mothers who did not participated in pregnant women conference were the identified determinants of the defaulter to full vaccination.

### Recommendation

The district health office should work on defaulters of vaccination by strengthening immunization service delivery through health communication activities by improving maternal knowledge and awareness of child vaccination with specific planning for children found in hard to reach area. Encouraging women’s participation in pregnant mother conferences and promoting institutional delivery to improve child vaccination through health extension program for further understanding of vaccine-preventable diseases and the importance of vaccines.

Health professionals should work on transferring appropriate health information to the child’s mothers/caretakers about the type of vaccine received, and the importance of childhood vaccination to prevent defaulting of the children from vaccination. Moreover, the researchers should conduct further studies on the topic using a prospective study design to investigate the detailed processes of the defaulter to full vaccination from basic roots through an ongoing process of child vaccination follow-up.

## Data Availability

All the data sets generated during and/or analyzed during the study are included in the study and available from the corresponding author upon reasonable request.

## **Abbreviations**

ANC: Antenatal Care
AOR: Adjusted Odds Ratio
COR: Crude Odds Ratio
DPT: Diphtheria Pertussis Tetanus
EDHS: Ethiopian Demographic Health Survey
EPI: Expanded Program on Immunization
Hib: Haemophilus influenzae type b
HepB: Hepatitis B
MCV: Measles Vaccine
PCV: Pneumococcal Conjugate Vaccine
SPSS: Statistical Package for Social Sciences
VPDs: Vaccine Preventable Disease
WHO: World Health Organization
